# Exercise and Alcohol Consumption: What We Know, What We Need to Know, and Why it is Important

**DOI:** 10.3389/fpsyt.2015.00156

**Published:** 2015-11-02

**Authors:** J. Leigh Leasure, Clayton Neighbors, Craig E. Henderson, Chelsie M. Young

**Affiliations:** ^1^Department of Psychology, University of Houston, Houston, TX, USA; ^2^Department of Psychology, Sam Houston State University, Huntsville, TX, USA

**Keywords:** alcohol, physical activity, drinking motives, reward, alcohol use disorder

## Abstract

Exercise provides a wealth of benefits to brain and body, and is regarded as a protective factor against disease. Protective factors tend to cluster together – that is, people who engage in one healthy behavior, such as exercise, also engage in other healthy behaviors, such as maintaining a nutritious diet and getting sufficient sleep. In contrast to exercise, alcohol consumption is not typically regarded as a health-promoting behavior, although moderate intake has been associated with a lower risk of cardiovascular disease. Surprisingly, several large, population-based studies have shown a positive association between physical activity and alcohol intake. The present review focuses on what is known about this relationship, including potential neural bases as well as moderating factors, and discusses important directions for further study, such as a more thorough characterization of people who both drink and exercise. We focus on ramifications for intervening with people who have alcohol use disorders, as exercise has been assessed as both a treatment and preventive measure, with mixed results. We believe that, in order for such interventions to be effective, clinical trials must distinguish treatment-seeking populations from non-treatment-seeking ones, as well as ensure that the use of exercise as a tool to decrease alcohol consumption is made explicit. We posit that a better understanding of the relationship between physical activity and alcohol intake will maximize intervention efforts by informing the design of clinical trials and research-driven prevention strategies, as well as enable individuals to make educated decisions about their health behaviors.

## Introduction

The relationship between alcohol and health is complex and multi-faceted. Despite its known health risks, less alcohol consumption does not necessarily translate to better health. Decades ago, a U-shaped curve was first used to depict the relationship between alcohol and mortality (1). This 10-year study showed that subjects with a moderate alcohol intake had the lowest mortality rates, suggesting that imbibing no alcohol at all carried the same risk level as heavy intake. It later became apparent that the effect of alcohol on mortality was largely due to a decrease in heart disease among moderate drinkers (2–4). This was surprising, given that alcohol can be cardiotoxic (5), and these findings spurred a great deal of further investigation into the health habits of moderate drinkers. A profile of moderate drinkers began to emerge, and this profile included physical activity, a nutritious diet, and other health-promoting behaviors (6). Although the veracity of the link between moderate alcohol use and better health has been called into question (7), decades of research on the health habits of moderate drinkers has focused interest on physical activity, resulting in the identification of a positive association between physical activity and alcohol intake.

Several large, population-based studies have shown that people who are physically active are also likely to be moderate drinkers (8, 9). Recently, exercise has begun to be implemented as an intervention for problematic alcohol use, including alcohol use disorders (AUDs). The present review focuses on the implications of the relationship between physical activity and alcohol consumption for the prevention and treatment of AUDs. We do not endeavor to provide exhaustive coverage of all extant studies of the relationship between alcohol consumption and exercise, as several excellent recent reviews have already done so (10–12). Instead, we focus on what is known about the relationship between physical activity and alcohol intake, gaps in the current knowledge, and implications for the nascent emergence of exercise-based interventions designed to decrease substance use. Throughout this review, we define “physical activity” as body movement that results in energy expenditure that surpasses baseline, and “exercise” as a subtype of physical activity that is intentionally performed in order to maintain or enhance fitness. We believe that a more complete understanding of the relationship between physical activity and alcohol intake will maximize intervention effects by informing the design of clinical trials and research-driven prevention strategies, as well as enable individuals to make educated decisions about their health behaviors.

## What We Know

### Considerable Evidence Indicates that Physical Activity and Alcohol Intake are Positively Related

The idea that physical activity and alcohol consumption are linked is not new. The relationship between drinking and athletic participation in college students has been studied for decades, and a number of these studies indicate heavier drinking in athletes compared to non-athlete peers [e.g., Ref. (13–15)]. A common explanation for these findings is that drinking goes along with athletic participation because teams celebrate victories (or commiserate losses) together, and that team association encourages alcohol consumption. However, the particular sport and/or competition level involved may play an important role in determining whether and how athletic participation influences substance use (16, 17).

Importantly, the link between physical activity and alcohol intake extends to college students who are not athletes (18–20) as well as to people who are not in college, and who do not play team sports. For an excellent recent review of studies documenting the link between activity and alcohol intake, see Ref. (10). One of the first indications of a link between activity and alcohol consumption outside of college sports was a national survey (21) that assessed responses from more than 40,000 American adults. They found that, compared to abstainers, moderate drinkers (4–7 drinks weekly for females; 8–14 for males) were twice as likely to be physically active. Moreover, as the intensity of activity increased, so did the strength of the association with alcohol consumption (21). More recently, French and colleagues (8) surveyed responses from over 230,000 American adults, and also found that drinking was associated with an increased probability of exercising. Interestingly, this relationship held up among heavy drinkers (>46 drinks in the past month for females; >76 for males).

Although these studies provide population-based evidence that alcohol and physical activity are positively related, they rely largely on subjects’ abilities to recall their typical alcohol intake and level of physical activity retrospectively, often for a significant period of time (e.g., the past 12 months or the past 30 days). A recent longitudinal study addressed this problem by having subjects keep daily diaries of their physical activity and alcohol intake over the course of 3 weeks (22). Analysis of the data showed a strong within-subject relationship such that individuals tended to drink more on the days they were more active. These data uphold the idea that the positive relationship between physical activity and alcohol consumption is not explained by errors in retrospective self-report.

To date, very little research has explored moderators of the association between alcohol use and physical activity, although a recent investigation found that age and gender are important factors. Lisha and colleagues (9) assessed over 30,000 survey responses from adults in the United States. They found an association between vigorous exercise and alcohol use that was strongest in respondents 50 years of age or younger. They also found that the association between moderate exercise and alcohol use was strongest in men. Furthermore, moderate physical activity in the past year was positively associated with alcohol use, particularly for males as compared to females. Similar results were obtained by Buscemi and colleagues (23), who found that physical activity was positively associated with drinking for men, but not for women. A recent study (24) conducted in Austria found no general link between activity and alcohol intake, but, interestingly, among individuals who reported consuming alcohol in the past week, men engaging in higher levels of physical activity also reported drinking more than men who engaged in moderate levels of physical activity.

### Biological Bases of the Exercise–Alcohol Use Relation

Consideration of the effects of alcohol and exercise on the brain is important for understanding why these two behaviors are linked. The positive association between exercise and alcohol intake may stem in part from the fact that both represent rewarding stimuli that invoke activity in the brain’s mesocorticolimbic pathway. This is a set of structures and connecting circuitry that extends from the ventral tegmental area in the midbrain (mesotelencephalon) to multiple forebrain structures, including cortical and limbic regions, such as the nucleus accumbens, amygdala, and prefrontal cortex. This reward circuitry evolved to respond to natural rewards that promote survival, such as sex, food, or exercise. For instance, exercise increases the release of dopamine and other monoamines [such as serotonin and norepinephrine; (25, 26)]. It also causes the release of endogenous opioids, such as endorphin (27, 28). Thus, exercise is a natural reward, but the alcohol we drink today is not. Humans figured out how to artificially stimulate neural reward circuitry with drugs (29) and alcohol represents a good example. Alcohol is a naturally occurring substance, and even non-human mammals will voluntarily consume it in the form of fermented organic material (30). However, humans began intentional fermentation in the Neolithic period (31), perhaps because they observed other species obtaining reward from its consumption. Intentional fermentation creates high alcohol-content beverages that represent highly rewarding, supraphysiological stimuli with addictive potential. Thus, both exercise and alcohol are capable of activating the mesocorticolimbic pathway, and have some overlapping neurochemical effects. Like all drugs of abuse, alcohol enhances dopamine activity in the mesocorticolimbic pathway (32). Moreover, both acute (33) and repeated (34) administration of alcohol increases endogenous opioids in this neural circuitry. Indeed, the rewarding effects of alcohol may result indirectly from its effects on endogenous opioid activity (35). It is largely because of this overlap in the effects of alcohol and exercise on dopamine and endogenous opioids that exercise has been suggested as a component of treatment programs for drug addiction. The idea is that exercise could partially activate the reward circuitry, possibly decreasing cravings via substitution (11, 12).

Another important circuit affected by both exercise and alcohol is the hypothalamic–pituitary–adrenal (HPA) axis, a key effector system involved in energy metabolism and stress responses. Exercise represents a challenge to homeostasis, but it is a predictable and voluntary form of “stress,” the practice of which seems to stabilize HPA axis function [for a review, see Ref. (36)]. The overall effect of this seems to help in the regulation of anxiety. Alcohol, too, impacts the HPA axis and while AUDs are associated with an increase in activity of the stress circuitry (37), in non-dependent individuals, a moderate dose of alcohol quells anxiety. To summarize, alcohol and exercise both have a broad range of effects on brain chemicals and circuitry, and some of these effects are overlapping. It is, therefore, conceivable that people who are not dependent on either alcohol or exercise may engage moderately in both on a regular basis in order to prolong positive affect.

### What We Can Learn from Animal Studies

Much of what we know about the neural effects of alcohol and exercise has been gleaned from animal studies, which enable researchers to control type, timing, intensity, and duration of exercise as well as the dose of alcohol and the circumstances under which it is available. Rodents are a good model for the study of both exercise and alcohol on the brain. Like humans, rodents find exercise rewarding, and will readily engage in wheel running [see Ref. (38), for a review], even in the wild (39). Moreover, rodents will consume alcohol (although there are strain and species differences in overall consumption), rendering them extremely useful for studying the neural effects of various amounts and patterns of alcohol consumption (40, 41).

Animal studies have also proven useful for studying the interaction between alcohol and exercise. Two recent reviews have exhaustively covered the extant literature on animal studies of the effects of exercise on alcohol and drug self-administration (11, 12). Both of them note that although there is strong pre-clinical evidence for exercise suppressing drug intake, alcohol seems to be an exception, in that there are mixed results concerning whether exercise increases, decreases, or has no effect upon alcohol intake.

Methodological differences may underlie these mixed findings. Importantly, the evidence for exercise decreasing alcohol consumption comes from studies in which both of these rewards were concurrently available. Some of the strongest pre-clinical evidence for a suppressive effect of exercise on alcohol intake comes from studies of mice that have concurrent access to both. Mice with access to a running wheel as well as a bottle of water and a bottle of alcohol drank less alcohol than sedentary mice with access to water and alcohol (42–44). This is a highly replicable finding, but also in contrast to studies in which alcohol and exercise are not concurrently available.

Another study in mice assessed the effects of repeated cycles of alcohol exposure (for 1 month), followed by wheel access with or without alcohol (45). Wheel access did not change alcohol intake, although it did decrease preference for it compared to water. By contrast, alcohol abstinence increased exercise distance and time, but this effect was reversed upon reintroduction of alcohol. Thus, this study provided evidence that drinking alcohol and exercising, both of which are rewarding, can serve as substitutes for each other.

In a study of an addiction-prone strain of rat (Lewis rats), running wheels were available, followed by access to alcohol without running wheels, and then alcohol withdrawal, with or without access to exercise wheels. When they were subsequently given access to alcohol again, rats that had access to running wheels during withdrawal drank more alcohol and preferred it more (compared to water) than they did compared to their own intake level during their first alcohol access (46).

To summarize, differences in experimental methodology may underlie the conflicting pre-clinical findings concerning the interaction of alcohol and exercise. A number of studies of the effect of exercise on alcohol intake in rodents have allowed concurrent access to both, and results of these studies indicate that exercise access decreases alcohol consumption. By contrast, when exercise and alcohol access are alternated, levels of both remain relatively stable. Finally, there is some evidence that exercise during alcohol withdrawal may increase subsequent alcohol consumption. These findings have important ramifications for clinical trials of exercise as an intervention for AUD.

### Summary

The positive association between physical activity and alcohol intake may initially appear paradoxical, as physical activity is regarded as a healthy behavior and excess alcohol use tends to be categorized as an unhealthy behavior. Yet the effect appears robust, as positive associations between drinking and physical activity have been found in both college students and the general population, across several measures of both alcohol use (e.g., frequency of drinking, quantity of drinks consumed per week/month, heavy episodic or binge drinking, peak drinks) and physical activity (e.g., exercise dependence questionnaire, international physical activity questionnaire; [see Ref. (9, 18–20, 22, 23, 47)]. Basic aspects of the relationship have begun to be studied, with a focus on demographics of exercising drinkers. Animal studies of the effect of exercise on alcohol intake have yielded mixed results, likely due to methodological differences.

Given that exercise has been proposed and is currently being investigated as an intervention for problem drinking, it is important to better understand the relationship between physical activity and alcohol intake. Keeping in mind the rewarding neural effects of both exercise and alcohol consumption provides a useful framework in which to view the association, as it highlights the importance of understanding the personality and motivational characteristics of exercising drinkers. A better understanding of the link between exercise and alcohol intake will inform clinical trials, and in the following section, we highlight gaps in the current understanding.

## What We Need to Know

### The Importance of Motivation in Understanding the Physical Activity and Alcohol Use Relation

As stated above, the question largely remains: who are these people that drink and exercise? In considering this question, we might ask more specifically about the motivations that may underlie both behaviors. Self-determination theory is among the most widely examined theories of human motivation and suggests that motivations for behavior range on a continuum from extrinsic motivation to intrinsic motivation (48, 49). Consistent with this perspective, Friederichs and colleagues (50) identified three clusters of motivations for engaging in physical activity: autonomous, controlled, and low motivation. Autonomous motivations are more consistent with intrinsic interests and well-integrated values, whereas controlled motivations are more consistent with extrinsic motivations and behaviors based on external contingencies, perceived expectations of others, and feelings of pressure. Friederichs and colleagues found distinctions between controlled and autonomous motivations using cluster analysis. Specifically, controlled motivations, relative to autonomous motivations, were associated with less education, higher body mass indices (BMIs), lower interest and enjoyment, less perceived competence, and lower ratings of effort, importance, value, and perceived choice.

Autonomous and controlled motivations have also been examined with respect to drinking. Although limited to college samples, the majority of the findings in this domain have suggested controlled motivations to be more strongly associated with drinking than autonomous motivations (51–53). Relatedly, Vallerand and colleagues’ (54, 55) Hierarchical Theory of Intrinsic and Extrinsic Motivation suggest that motivations can be viewed from varying levels of generality (i.e., global, contextual, and situational) and that motivations are correlated across levels. Thus, an individual who is generally extrinsically motivated is likely to be extrinsically motivated to engage in specific domains such as physical activity and drinking. Combining self-determination and hierarchical theories with regard to physical activity and drinking may help us better understand why drinking and physical activity are related from a motivational perspective and may further help distinguish multiple relationship types among these domains. For example, at the global level, individuals who are extrinsically motivated may engage in drinking and exercise for extrinsic reasons. In this case, extrinsic motivation might serve as a third variable accounting for the relationship between both activities. That is, drinking and physical activity at this level may not be causally associated, but instead reflect a general motivational tendency that accounts for both activities.

At the contextual level, we might consider motivations for drinking and motivations for exercise and the extent to which these motivations overlap. Four general motives for drinking have been identified and extensively studied (56–58). These include social, enhancement, coping, and conformity motives (59). Social motives for drinking describe drinking in order to have fun with others and to facilitate drinking in social situations. Enhancement motives, often highly correlated with social motives, refer to drinking as a means of enhancing affect or feeling good. Coping motives refer to drinking as a means of escape or avoidance of negative affect. Finally, conformity motives, which are the least commonly endorsed, refer to drinking in order to avoid rejection from others. These motivations for drinking may overlap with some of the same motivations individuals have for engaging in physical activity and exercise. For example, both psychological health and interpersonal motivations have been identified for engaging in exercise (60).

Although there are extensive literatures examining drinking motives and exercise motives, to our knowledge no previous research has directly examined joint motivations between drinking and exercise. Here, we propose at least four possible motives: work hard play hard, celebration, body image, and guilt. As elaborated below, work hard play hard and celebration motives would place exercise as the antecedent of alcohol consumption whereas body image and guilt motives would place alcohol consumption as the antecedent of exercise.

The work hard play hard motivation has been suggested as an explanation for higher rates of drinking among college student athletes (61, 62). As indicated by the label, the idea is that engaging in hard work, especially hard physical work, has hedonic reward. The relatively large demands on student athletes and an associated “work hard–play hard” attitude has been offered as one of the contributors to higher drinking rates among student athletes versus other students (14, 63). More broadly, the association between alcohol and sports in the U.S. has also been attributed, at least in part, to the idea that working hard, especially physically intensive effort, goes hand in hand with drinking (64). The implicit notion is that working hard, especially physically, earns the right to consume alcohol or engage in other indulging activities.

**Figure 1:**
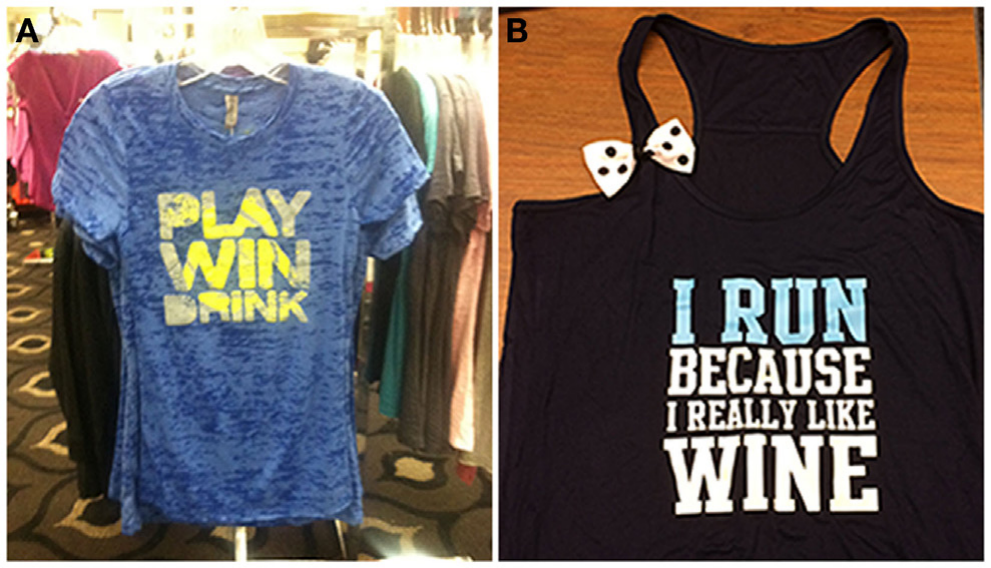
**The celebration motive (A) and body image motive (B) are illustrated by these items of clothing**.

Celebratory drinking is often done to commemorate special occasions, such as academic or professional achievements, milestones, birthdays, or holidays. Importantly, celebration drinking may also be associated with physical achievements, such as completing a race, meeting physical goals for exercise, or winning competitive physical competitions (see Figure 1). The relationship between exercise and celebratory drinking is particularly interesting because an athletic victory may be the occasion that spurs the drinking, as has been shown in college athletes (61, 62) (see Figure 1).

Body image and exercise are conceptually entwined with one another in both adaptive and potentially harmful ways. For instance, a recent meta-analysis showed significant improvements in body image following exercise interventions (65), indicating exercise may function as a potential tool for addressing low body image. However, excessive exercise to improve body image is also a defining feature of eating disorders, especially for men (66). Body dissatisfaction has been linked to higher levels of drinking, with a potential mechanism for this relation existing through attempts to reduce negative affect (67). Recently, popular culture has coined the term “drunkorexia” to describe skipping meals and/or engaging in excessive exercise in order to “bank” calories for future binge drinking. Emerging research on this topic suggests that the underlying motive for such behavior is avoiding weight gain and that it is more prevalent in females than males (68). A recent study examining “drunkorexia” found that women who use exercise as a compensatory behavior drank more alcohol, engaged in more heavy episodic drinking, and experienced more alcohol-related problems than women who do not use exercise as a compensatory behavior; however, there were no differences in drinking frequency between the groups (69). Additionally, women who reported using exercise as a compensatory behavior more strongly endorsed both drinking and exercise motives and scored higher on measures of impulsivity, body dissatisfaction, and dietary restraint. Thus, a better understanding of motivations underlying associations between exercise and alcohol use is needed.

Guilt is a negative, moral-focused, self-conscious emotion that is associated with behavior change (70). Specifically, guilt is negative emotion regarding one’s engagement in a certain behavior, such as excessive drinking [“I should not have drunk that much last night”; (71, 72)]. When individuals experience guilt, they may feel tense or regretful and desire to alleviate this unpleasant feeling by making amends for their behavior; for example, by engaging in a health-promoting behavior such as exercise. Thus, guilt is associated with positive, responsible behaviors, working toward improving one’s self, and compensating for past mistakes (e.g., drinking too much), and as such is theorized to be an adaptive emotion (72).

### Studying Personality Characteristics, Social Factors, and Comorbidities May Explicate the Relationship Between Physical Activity and Alcohol Use

Personality characteristics influence both exercise behaviors and drinking behaviors. For example, past work has shown that highly conscientious individuals are more likely to exercise and less likely to drink heavily whereas extraverted individuals are more likely to drink alcohol and engage in other unhealthy behaviors (73). Personality characteristics likely also influence the relationship between physical activity and alcohol intake. To illustrate, more extraverted individuals may be more likely to find themselves in situations where alcohol is present and may also be more likely to exercise with others. However, the personality characteristics of those who both drink and exercise are almost completely unstudied. A recent study found that the relationship between exercise and alcohol intake was strongest among those who scored highest on indices of impulsivity, including positive urgency and sensation seeking (47). Other personality constructs should be investigated as this will help to distinguish what populations are most likely to benefit from exercise-based interventions.

Social factors may also account for the link between exercise and alcohol use. Previous research has shown that shy and socially anxious individuals tend to drink less (in quantity and frequency) than their non-shy or non-socially anxious counterparts (74–78). This negative association between shyness/social anxiety and drinking is thought to exist in part because shy/socially anxious individuals are less likely to find themselves in social situations where drinking occurs. Similarly, individuals higher in social anxiety or shyness may also be less inclined to exercise around others (e.g., in a gym). Research has found that social physique anxiety, a form of self-presentation anxiety specific to one’s appearance (79), was negatively associated with self-reported exercise (80). Therefore, anxiety may prevent individuals from engaging in physical activity because they desire to avoid contexts in which they are evaluated based on their physical appearance. Relatedly, college-aged women higher in social physique anxiety were found to be more likely to exercise in private rather than in public settings (81). Thus, individuals higher in shyness, social anxiety, or specifically social physique anxiety may choose either not to exercise or to exercise alone, removing the social and potentially evaluative aspect of the gym. Social physique anxiety is also associated with greater extrinsic reasons for exercising such as for social interaction purposes and body appearance reasons (82), further indicating that motivation is an important factor to consider regarding the link between alcohol and exercise (see The Importance of Motivation in Understanding the Physical Activity and Alcohol Use Relation).

Finally, examining comorbidities may be useful for characterizing people who both drink and exercise, as they may be distinguished by behaviors that they do *not* engage in. Research has supported a health cluster delineation such that healthy behaviors tend to cluster within individuals and, similarly, unhealthy behaviors also tend to cluster (83–89). However, some research has shown that frequent exercisers tend to drink more frequently, consume greater quantities of alcohol, and engage in more heavy drinking episodes, but are less likely to smoke compared to those who exercise infrequently (18). In other words, alcohol use and physical activity are likely to co-occur; however, individuals who both drink moderately and exercise are often less likely to also smoke. In this vein, several studies have found that physical activity and smoking tended to be negatively associated (90–92). Research also suggests that individuals who drink and exercise can be distinguished from individuals who engage in several unhealthy behaviors. For instance, a survey of high school students found that individuals who engaged in lower levels of physical activity were more likely to smoke cigarettes, use marijuana, and eat less fruits and vegetables (93). Another study with an Australian young adult sample found that smoking, drinking excessively, and eating unhealthily were clustered among the sample overall and that these behaviors also clustered with physical inactivity among women (94).

Recent estimates suggest that the past-year prevalence rate of AUDs is 13.9%, with lifetime prevalence rates of AUDs at 29.1% (95). Research has also examined comorbidities between physical activity, alcohol and other substance use disorders, and psychiatric disorders. Individuals who engage in physical activity on a regular basis tend to have a lower likelihood of psychiatric comorbidities, such as major depressive disorder, agoraphobia, generalized anxiety disorder, specific phobia, and social phobia (96, 97). However, regular physical activity was found to be unrelated to substance use disorders (96). Conversely, individuals with substance use disorders have a greater likelihood of also having psychiatric disorders such as major depressive disorder, bipolar I disorder, borderline personality disorder, antisocial personality disorder, panic disorder, specific phobia, and generalized anxiety disorder (95, 98, 99). Thus, physical activity is associated with a lower likelihood of psychiatric disorders, whereas AUDs are associated with a greater likelihood of psychiatric disorders. The current literature found no association between physical activity and substance use disorders, including alcohol dependence. Further research should examine comorbidities among AUDs, psychiatric disorders, and physical activity to better understand how to target interventions for these individuals.

### The College Years are a Developmentally Sensitive Period During which the Relationship Between Physical Activity and Alcohol Consumption should be Studied

The research of Conroy and colleagues is significant, as it is the first study to examine the within-subject relation between exercise and alcohol use, and the findings are clinically meaningful, as it suggests that these behaviors are linked at the level of the individual person (22). As a result, and as we discuss in more detail below, in order for exercise interventions designed to decrease alcohol use to be effective, clinical researchers must somehow decouple these behaviors. However, as with all research, the generalizability of the findings should be examined through replication. Furthermore, although studying the relation over such a large age range (19–89) is a strength of the study, it collapses participants across potentially important developmental periods.

For instance, college students are an important subgroup in which the relation should be examined in more detail. Most college students are between the ages of 18 and 24, which constitutes late adolescence (100), and is a critical developmental period in which health behavior patterns emerge (101) and often become solidified in later adulthood, thereby affecting life-long health (102). Over 16% of individuals between the ages of 18 and 29 qualify for an AUD (103), with some research suggesting that aspects of the college experience itself leads to greater alcohol use. For instance, studies have shown that college students are not only more likely to consume alcohol than their non-college attending peers (104), but are also more likely to exhibit problem drinking behaviors (105). Moreover, up to 50% of college students describe themselves as physically inactive (106). Clearly, interventions that could modify both problematic alcohol use and physical inactivity among college students would be valuable, but in order to be maximally effective, we must first understand the exercise–alcohol use relation in this developmental period.

Intriguingly, it is possible that the positive relationship between exercise and alcohol intake shifts from a negative one early in adolescence to a positive one in later adolescence. Analysis of over 650,000 Monitoring The Future questionnaires completed by 8th, 10th, and 12th grade students between 1991 and 2009 showed that exercise was negatively related to alcohol use (107). Yet, as described in Section “Considerable Evidence Indicates that Physical Activity and Alcohol Intake Are Positively Related,” a number of studies document a positive relationship between exercise and alcohol consumption in college students and in the general population. Thus, while the field will benefit from studying this relationship throughout the lifespan, the period of late adolescence encompassed within the college years may represent a unique transitional period that merits particular attention.

## Why it is Important

### Exercise Interventions with Non-Treatment-Seeking Populations

As indicated above, since both exercise and drinking alcohol are rewarding, experiencing one could influence engagement in the other. According to Conroy et al., these behaviors are functionally coupled (22). This suggests that exercise may be of limited utility as an intervention method, and, at worst, may have iatrogenic effects. On the other hand, the overlapping effects of alcohol and exercise on neural reward circuitry, as well as theoretical propositions from behavioral economics such as the reinforcement potential of substance-free activities (SFAs) suggest that exercise may partially compete with alcohol use.

The value of SFAs has also been demonstrated empirically (108). Much of this work has been done with college students who were not seeking treatment. Corriea and colleagues (109) found that heavy drinking college students reported lower frequency and enjoyment of SFAs competing with alcohol use (e.g., hiking, art projects, pleasure reading) in comparison to students who were not heavy drinkers. The reinforcement potential of alternative alcohol-free behaviors has been intentionally harnessed in intervention studies designed to increase SFAs as a means to decrease substance use. Murphy and colleagues (110, 111) have linked SFA sessions focused on increasing engagement in academic and constructive campus activities, including exercise, with brief motivational interventions and achieved promising results. With respect to exercise as a specific SFA, Murphy et al. (112) randomized 60 heavy drinking college students to an exercise intervention, a meditation intervention, or a no-treatment control group and found that the greatest reductions in alcohol use were realized with the exercise intervention. In a second study, Correia et al. (113) randomized 105 college student substance users (primarily drinkers) to a condition in which they were instructed to reduce their substance use, one in which they were instructed to increase their physical and creative activity, or a control group in which they received no instructions. These researchers found that in addition to increasing their physical and creative activities, the increased activity group spontaneously reduced their drinking to a greater extent than the control group.

Recognizing the potential of exercise-based interventions, Weinstock and colleagues (114, 115) have initiated a program of research designed to decrease hazardous drinking among college students using exercise-based interventions. These studies have remedied some of the limitations of the seminal Murphy et al. trial, namely exclusive self-report of exercise behaviors and high attrition. They have also specifically targeted exercise behaviors in contrast to Correia et al., which instructed participants more generally to increase their physical and creative activity. In addition, in light of the low adherence and high attrition common in general exercise interventions (116, 117), Weinstock and colleagues have integrated contingency management (CM) and motivational enhancement therapy (MET) to increase participants’ extrinsic and intrinsic motivations to exercise. Weinstock et al. (114) randomized 31 participants to an MET + CM 8-week exercise-based intervention or one session of MET focused on increasing exercise. Participants were not receiving alcohol treatment, and by a study exclusion criterion did not express a desire to receive it. They were sedentary (exercising <2 days per week within the last 2 months), received scores of 8 or higher on the Alcohol Use Disorders Identification Test (AUDIT), indicating hazardous drinking (118) and reported four or more heavy drinking episodes in the past 2 months. Weinstock et al. (115) randomized 70 participants to the same MET + CM condition (but included two instead of one MET session) or MET + Exercise Contracting. In the latter condition, participants completed weekly exercise contracts, but, unlike the MET + CM condition, their exercise behavior was not reinforced. In both studies, participants exercised on their own without supervision and provided validation of having exercised using sign-in logs for exercise classes or brief smartphone videos of their exercise sessions. Exercise was individualized, based on agreements made between participants and interventionists during their weekly contracting sessions. The same inclusion/exclusion criteria were employed as in Weinstock et al. (114). Whereas these studies found that the MET + CM exercise intervention increased students’ exercise more than comparison treatments at a 2-month post-intervention assessment and decreased alcohol use across treatment conditions (115), they have not resulted in treatment differences in alcohol use. However, Weinstock et al. (114) found a moderate effect size (*d* = 0.49) favoring the MET + CM condition for reducing the number of drinking days.

To summarize, interventions developed to increase SFAs to compete with alcohol use and to enhance motivation to exercise through reinforcement have succeeded in increasing exercise behavior (both frequency and volume) during the period participants receive the intervention. However, findings have revealed that the increased exercise has not had the desired impact of decreasing alcohol use.

### Exercise as an Adjunctive Treatment for Alcohol Dependence

The literature on the use of exercise as an adjunctive therapy in conjunction with alcohol treatment for alcohol-dependent individuals has been studied more frequently, and appears to be more consistent. This work is summarized in a recent meta-analysis conducted by Geisen et al. (119). The authors synthesized data from 14 randomized controlled trials (RCTs) addressing exercise interventions among individuals receiving treatment for an AUD. Inclusion criteria consisted of individuals with a clinically indicated AUD or labeled as problem/harmful drinkers and excluded: (1) subclinical populations, which Geisen et al. define as “heavy/hazardous drinkers” or “social drinkers”; (2) youths under 20 years of age; (3) persistence of AUD for less than 5 years in duration; and (4) studies in which exercise did not constitute the central component of the intervention (i.e., lifestyle modification programs). Notably, only five of these trials examined the impact of the exercise intervention on alcohol use. One might assume the trials that did not report alcohol outcomes may likely have found non-significant results given Geisen et al.’s inclusion criteria and, thus, suffer from the commonly occurring file-drawer problem (120). Results did not include a combined effect size, but were instead summarized by noting that three studies showed that the intervention group demonstrated greater decreases than a comparison group in craving (121), abstinence rates (122), and amount and frequency of alcohol use (123). Two studies (124, 125) found no differences between experimental and comparison groups, although across treatments participants decreased their alcohol use over time (see Table 1).

**Table 1:** **Descriptive information for the five exercise intervention studies in the current review**.

Study	Sample	Intervention conditions	Alcohol outcome	Effect size^a^
Murphy et al. (112)	Undergraduate college students attending a large state university	(1) Aerobic exercise (running)	Journal entries reflecting alcohol consumption	Effect size could not be calculated from information provided
		(2) Meditation		
		(3) Control		
Correia et al. (113)	Undergraduate college students attending a large private university	(1) Instruction to reduce substance use	DDQ	Alcohol use days (*d* = 0.22)
		(2) Instruction to increase physical and creative activity		Total standard drinks (*d* = 0.26)
		(3) Control		
Weinstock et al. (114)	Undergraduate college students attending a moderate-sized state university	(1) MET	TLFB for alcohol use	Alcohol use days (*d* = 0.48)
		(2) MET + CM		Total standard drinks (*d* = 0.15)
Weinstock et al. (115)	Undergraduate college students attending a moderate-sized state university	(1) MET + Exercise Contracts	TLFB for alcohol use	Binge drinking (*d* = 0.01)
		(2) MET + CM		Total standard drinks (*d* = 0.18)
Brown et al. (123)	Alcohol-dependent adults either: attending a day-treatment program or living in the community	(1) Aerobic exercise	TLFB for alcohol use	Drinking days rate (Ratio = 0.27)
		(2) Brief advice to exercise		Heavy drinking days rate (Ratio = 0.54)

*CM, contingency management; DDQ, daily drinking questionnaire; MET, motivational enhancement therapy; TLFB, timeline follow-back*.

*^a^Effect sizes reflect differences between conditions. In three-group designs, the effect size reflects the difference between the physical activity condition and control group*.

Among the studies ranked higher in methodological quality by Geisen et al. were two studies reporting alcohol outcomes, a doctoral dissertation, Donaghy (124) and Brown et al. (123). Because the Donaghy study is an unpublished dissertation, we will focus on Brown et al.’s findings here, but interested readers are encouraged to see Geisen et al.’s meta-analysis for more detail. Brown and colleagues (123) randomly assigned 49 alcohol-dependent individuals (based on DSM-IV-TR diagnosis) in an outpatient day-treatment setting to either the standard treatment provided at the facility with advice to exercise, or the standard treatment plus a 12-week group aerobic exercise intervention. Participants in the exercise intervention condition engaged in a once-weekly, 20–40 min supervised exercise session and were advised to engage in 2–3 non-supervised exercise sessions. They also received weekly group-based behavioral interventions focused on improving their physical fitness. In addition, their exercise was incentivized with a CM “fish bowl” intervention similar to that implemented in Weinstock’s and colleagues studies summarized above, although the reinforcement schedules differed. Findings indicated that participants receiving the exercise intervention reported lower levels of alcohol use frequency and heavy drinking episodes at the end of treatment (*p*’s < 0.001), but these gains were not sustained through the 12-week follow-up. The researchers observed a dose–response relation in both conditions in which exercise was negatively related to alcohol frequency at both time points (*p*’s < 0.001) and to heavy drinking episodes at the 12-week follow-up (*p* < 0.001).

### Treatment Seeking: A Key Variable in Explaining Mixed Findings from Exercise Intervention Trials

In summary, the literature is decidedly mixed with regard to the effectiveness of exercise-based interventions targeting problematic alcohol use. Here, we attempt to resolve some of the discrepancies in the literature to date and provide direction for future randomized clinical trials. Given sample heterogeneity, it is difficult to draw conclusions regarding the efficacy of interventions by severity of alcohol use. In other words, the literature does not provide a clear-cut distinction between prevention and treatment samples, as studies implementing exercise interventions as preventive measures (e.g., hazardous drinking among college students) have enrolled participants with extensive drinking histories who presumably may be in need of more concentrated treatment [i.e., Ref. (114, 115)].

Yet, in terms of brain functioning, distinctions, such as those between treatment seekers and non-treatment seekers, may indeed be useful. Research has demonstrated that the alcohol-dependent brain is different from the non-alcohol-dependent brain, both chemically and structurally speaking. Simply put, a large body of research documents lasting AUD-induced brain changes in which reward circuitry is desensitized to reward and stress circuitry is overactive [for reviews, see Ref. (126–129)]. The distinctions that can be drawn at the level of brain functioning may not clearly translate upward to behavior-based measures such as screening measures and diagnostic interviews (130). Nonetheless, separating dependent from non-dependent populations might produce a more robust effect of exercise on the reduction of drinking behavior. Relatedly, treatment-seeking versus non-treatment-seeking individuals may provide disparate responses to intervention efforts.

In the following content, we contrast the work of Weinstock and colleagues and Brown and colleagues for the following reasons: (1) they incorporate many of the methodological features that have become the field’s standards in contemporary alcohol treatment research (131) and (2) they test empirically supported interventions for participants with AUDs (MET, CM). Therefore, these two research programs provide the field’s most rigorous empirical evidence we have to date regarding the effectiveness of exercise-based interventions on alcohol outcomes. Notably, Brown et al.’s work (123, 132) has indicated that these interventions are effective, and although Weinstock et al.’s work (114, 115) has suggested that exercise interventions increase participants’ exercise behaviors, these differences have not translated to alcohol outcomes.

A primary distinction between these groups’ research programs is whether or not the samples were seeking treatment when they were enrolled in the study. Because college students frequently do not identify heavy drinking as a concern, and seldom seek treatment voluntarily (133, 134), Weinstock and colleagues have intentionally masked a connection between exercise and alcohol use and developed interventions for students who were not seeking intervention. The argument being that “offering interventions for heavy drinking that do not stigmatize or require an individual to see a mental health professional may increase the utility and acceptability of the intervention and ultimately increase the number of individuals effectively treated” (135) (p. 539). On the other hand, there was no way to mask the connection between exercise and alcohol use in Brown and colleagues’ trials because the participants were already enrolled in treatment to decrease their substance use. As we argue below, differences between the samples in terms of whether they were seeking treatment when they were enrolled in the exercise intervention may also translate to whether an explicit connection is made for participants between their exercise and alcohol use.

### Conclusion and Recommendations Regarding Future Alcohol Interventions Incorporating Exercise

**Figure 2:**
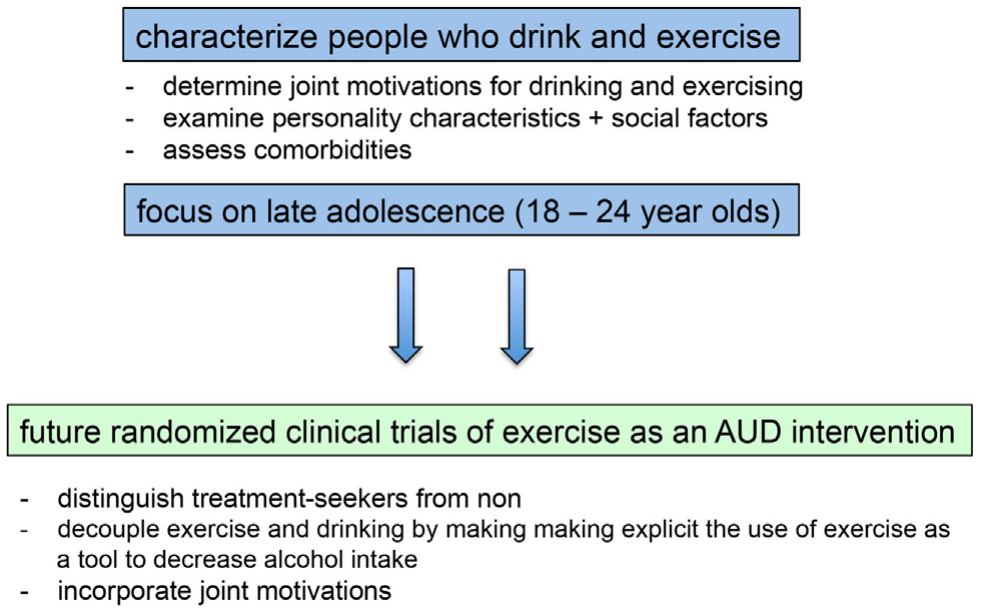
**Filling in existing knowledge gaps will inform the development and implementation of future randomized clinical trials of exercise as an intervention for alcohol use disorders**.

The preponderance of the evidence from correlational and epidemiological studies examining the relation between exercise and alcohol use suggests that exercise is positively related to alcohol use, and research to date does not suggest that this relation is necessarily harmful to health in non-dependent individuals [but see Ref. (136)]. Results from these studies present a conundrum for researchers motivated to develop interventions simultaneously targeting these two important health behaviors. We contend that this association must be taken into account if exercise is to be used successfully as an intervention for AUD. Here, we suggest several ways in which this issue can be addressed in future studies (see Figure 2).

Results from the limited research examining within-subject relations indicates that physical activity and alcohol use are “functionally coupled,” meaning that the association reflects a process that varies from day-to-day, such that individuals drink more on days they exercise more (22). Conroy et al. suggest that such interventions may need to incorporate “a functional substitute … to decouple them or reverse the direction of their coupling” (p. 6). Results from intervention trials suggest that this may make the positive association between exercise and alcohol use explicit. For example, if activity (including mental activity) is used as a means by which to consciously attenuate drinking, alcohol intake decreases (113). Thus, *intent* may be an important aspect of the link between exercise and drinking. In the case of using exercise as a means to prevent or decrease drinking in non-AUDs, exercise could be used with conscious intent as a means to engage in pleasurable activities that compete with substance use. In the case of using exercise as a treatment for AUDs, conscious intent could be used to cement exercise as a means by which to reduce craving, provide reward, or reduce anxiety.

Another means by which to increase the success of exercise as an AUD intervention is to clearly distinguish treatment seekers from non-treatment seekers. Indeed, among participants seeking treatment, when the association between exercise and alcohol use is inherent in the intervention, the results of intervention trials in recent years have been positive. That said, more trials are needed, as the research base is small. Intervention with participants who are not seeking treatment is more complicated, and trials in recent years have been designed with the implicit assumption that getting participants to exercise more would spill over to have a positive impact on reducing their alcohol use. And, the results from early intervention studies, and those from studies incorporating exercise in a more general class of SFAs, have been positive. However, recent studies directly targeting exercise as a means for decreasing alcohol use have not had the desired impact.

What would an intervention for participants *not* seeking treatment for alcohol use (e.g., college students) look like? We are encouraged by the results of intervention research with college students using personalized feedback interventions (PFIs) with effect sizes in the moderate to large range (137). PFIs could also be incorporated in the context of an exercise intervention, in which participants are queried about their recent exercise behavior and alcohol use, educated regarding the positive association between exercise and alcohol use, and provided reinforcement for achieving their goals relating to both of these behaviors.

Finally, a better understanding of motives should be integrated in intervention contexts. For example, interventions might incorporate feedback regarding the specific motives between physical activity and drinking for each individual and consider whether there are positive changes that could be made based on the idiographic connections. This coupling should be incorporated into intervention efforts by a thorough assessment of motivations linking these behaviors. For instance, if a person is identified as one motivated by the work hard play hard motive, then an effective intervention might include protective behavioral strategies in which the subject is encouraged to drink a glass of water between alcoholic beverages. By contrast, if a person is motivated by body image concerns and is calorie banking to be able to compensate for the calories in alcohol, the intervention might include nutritional information as well as a brief screening or potential referral for eating-disorder assessment. Moreover, if future research suggests that one motivation is more prevalent in one gender than another, this could indicate the need for gender-focused intervention strategies. Ultimately, the ability to tailor intervention efforts to individual motives requires more research and a better understanding of individuals who both drink and are physically active.

## Conflict of Interest Statement

The authors declare that the research was conducted in the absence of any commercial or financial relationships that could be construed as a potential conflict of interest.
